# Supplementary material for: The Systems Biology Research Tool: evolvable open-source software
Source: BMC Syst Biol. 2008 Jun 29;2:55. doi: 10.1186/1752-0509-2-55 (PMC2446383; doi:10.1186/1752-0509-2-55)
Supplement: Additional file 1 — SBRT Archive. An archive of the current version of the Systems Biology Research Tool. [file 1752-0509-2-55-S1.zip › sbrt-1.4.0/doc/developers_guide/api/sbrt/kernel/fba/comp/WwFluxomeReducer.html]

WwFluxomeReducer


|  |  |  |
| --- | --- | --- |
| **Overview** \| **Package** \| **Class** \| **Use** \| **Tree** \| **Deprecated** \| **Index** \| **Help** | |  |
| **PREV CLASS**   NEXT CLASS | **FRAMES**    **NO FRAMES**     **All Classes** |  |
| SUMMARY: NESTED \| FIELD \| CONSTR \| METHOD | DETAIL: FIELD \| CONSTR \| METHOD |  |


---


## sbrt.kernel.fba.comp Class WwFluxomeReducer

```
java.lang.Object
  sbrt.kernel.fba.comp.WwFluxomeReducer
```

**All Implemented Interfaces:**: FluxomeReducer<IrrevRxnFluxome>

---

``` public class WwFluxomeReducer extends java.lang.Object implements FluxomeReducer<IrrevRxnFluxome> ```

This class is used to reduce the size of fluxomes for the purpose of
identifying the steady state cycles (Type III extreme
pathways) they contain.
It is an implementation of the algorithm described by Wright and Wagner (WW).

**Author:**
:   This class was written and documented by Jeremiah Wright while in the
    Wagner lab.

---

**Constructor Summary**

`WwFluxomeReducer(ProgramSolver<java.lang.Object,java.lang.Object> solver)`
  
          Constructs a fluxome reducer from the provided linear
program solver.


| **Method Summary** | |
| --- | --- |
| `ProgressMonitor` | `getProgress()`             Returns a progress monitor for this fluxome reducer. |
| `IrrevRxnFluxome` | `makeFluxome(IrrevRxnFluxome fluxome)`             Returns the sub-fluxome resulting from linear programming. |

**Methods inherited from class java.lang.Object**

`clone, equals, finalize, getClass, hashCode, notify, notifyAll, toString, wait, wait, wait`

**Constructor Detail**

### WwFluxomeReducer

```
public WwFluxomeReducer(ProgramSolver<java.lang.Object,java.lang.Object> solver)
```

:   Constructs a fluxome reducer from the provided linear
    program solver.

    **Parameters:**: `solver` - the linear program solver with which all optimizations will be computed.


**Method Detail**

### getProgress

```
public ProgressMonitor getProgress()
```

:   Returns a progress monitor for this fluxome reducer.

    :   **Specified by:**: `getProgress` in interface `FluxomeReducer<IrrevRxnFluxome>`
    :   **Returns:**: a progress monitor for this fluxome reducer.

---


### makeFluxome

```
public IrrevRxnFluxome makeFluxome(IrrevRxnFluxome fluxome)
```

:   Returns the sub-fluxome resulting from linear programming.

    :   **Specified by:**: `makeFluxome` in interface `FluxomeReducer<IrrevRxnFluxome>`
    :   **Parameters:**: `fluxome` - the fluxome for which a sub-fluxome will be returned. **Returns:**: the sub-fluxome resulting from linear programming.


---


|  |  |  |
| --- | --- | --- |
| **Overview** \| **Package** \| **Class** \| **Use** \| **Tree** \| **Deprecated** \| **Index** \| **Help** | |  |
| **PREV CLASS**   NEXT CLASS | **FRAMES**    **NO FRAMES**     **All Classes** |  |
| SUMMARY: NESTED \| FIELD \| CONSTR \| METHOD | DETAIL: FIELD \| CONSTR \| METHOD |  |


---
